# HoLEP using a 21 Fr internal urethrotomy sheath, a feasible alternative to MiLEP in resource-limited settings

**DOI:** 10.1007/s00345-026-06554-3

**Published:** 2026-06-27

**Authors:** Aykut Aykaç, Coşkun Kaya, Mustafa Sungur, Mehmet Erhan Aydın

**Affiliations:** 1 Department of Urology, University of Health Sciences Eskişehir City Health Application and Research Center, Eskisehir, Turkey; 2 Eskişehir City Hospital, 71 Evler Neighborhood, Çavdarlar Street, Odunpazari, Eskisehir Turkey

**Keywords:** Benign prostatic hyperplasia, HoLEP, Suprapubic drainage, Lower urinary tract symptoms, Resource-limited settings

## Abstract

**Purpose:**

Holmium laser enucleation of the prostate (HoLEP) is an effective, size-independent surgical treatment for benign prostatic hyperplasia; however, the use of large-caliber resectoscope sheaths may increase urethral trauma. Dedicated minimally invasive laser enucleation of the prostate (MiLEP) systems aim to reduce this risk but are not universally available. This study aimed to evaluate whether a modified HoLEP technique using a 21 Fr internal urethrotomy sheath (mLC-HoLEP) technique performed with commonly available endourological instruments provides comparable short-term functional and safety outcomes to conventional HoLEP in settings without access to dedicated MiLEP systems.

**Methods:**

This single-center retrospective cohort study included patients who underwent standard HoLEP (*n* = 48) or mLC-HoLEP (*n* = 47) between January 2021 and May 2023. The mLC-HoLEP technique utilized a 21 Fr internal urethrotomy sheath combined with passive bladder drainage via a 16 G suprapubic cannula, followed by standard morcellation with a 26 Fr morcelloscope. Groups were matched using propensity score matching. Perioperative parameters, functional outcomes (IPSS, Qmax, postvoid residual urine), irrigation volume, and early postoperative complications were compared.

**Results:**

Baseline demographic and preoperative characteristics were comparable between groups. Operative time, enucleation time, morcellation time, hemoglobin drop, and specimen weight showed no significant differences. Total irrigation volume was significantly lower in the mLC-HoLEP group (*p* < 0.001). Postoperative IPSS, Qmax, and residual urine volumes were similar between groups. Early postoperative complications and Clavien–Dindo complication grades did not differ significantly. No complications related to suprapubic drainage were observed.

**Conclusion:**

Modified low-caliber HoLEP performed with commonly available endourological instruments offers comparable functional efficacy and safety to conventional HoLEP while significantly reducing irrigation volume, representing a feasible resource-adapted technical modification that eliminates the need for additional equipment acquisition, with potential practical advantages particularly in resource-limited settings.

## Introduction

Benign prostatic hyperplasia (BPH) is one of the most common causes of lower urinary tract symptoms (LUTS) in aging men and significantly impairs quality of life. Surgical treatment is required in patients who do not respond adequately to pharmacological therapy [1]. Although transurethral resection of the prostate (TURP) has long been considered the standard surgical approach, holmium laser enucleation of the prostate (HoLEP) has emerged as a minimally invasive alternative with proven efficacy across a wide range of prostate volumes and is currently recommended in guidelines even for large prostates [2–5].

The main advantages of HoLEP include its high tissue removal efficiency independent of prostate size, low bleeding risk, short catheterization and hospital stay, and its safe applicability in patients receiving anticoagulant therapy [6]. However, standard HoLEP is typically performed using 26–28 Fr resectoscope sheaths, which may increase the risk of mechanical urethral trauma and subsequent stricture formation. Reported urethral stricture rates following HoLEP range between 2% and 5% in the literature [7].

In recent years, techniques aimed at reducing urethral trauma through miniaturization of surgical instruments have been developed. Minimally invasive laser enucleation of the prostate (MiLEP) is performed using dedicated miniaturized instruments with diameters of 18.5–22 Fr and has been associated with a reduced need for urethral dilation and lower rates of early transient urinary incontinence compared with standard HoLEP [3, 8, 9]. However, the specialized resectoscopes, fiberoptic nephroscopes, and continuous-flow irrigation–outflow systems required for MiLEP are not interchangeable with standard HoLEP equipment and require separate procurement, representing an incremental capital investment that may limit accessibility in resource-constrained settings. In contrast, our technique relies exclusively on instruments already available in virtually all endourology units, constituting a resource-adapted technical modification that eliminates the need for additional equipment acquisition. This limitation substantially restricts its clinical applicability, particularly in resource-limited settings.

At this point, the fundamental clinical question is whether similar clinical advantages can be achieved using smaller-caliber sheaths without the need for expensive and dedicated MiLEP equipment.

In this study, we evaluated the clinical outcomes of a modified low-caliber HoLEP (mLC-HoLEP) technique performed using a commonly available 21 Fr internal urethrotomy sheath combined with suprapubic bladder drainage in a center without access to original MiLEP equipment.

The aim of the study was to compare the short-term functional outcomes and complication profile of this modified technique with those of standard HoLEP in order to determine whether it represents a safe, effective, and accessible alternative.

## Materials and methods

### Study design and patient selection

This single-center retrospective cohort study evaluated the outcomes of patients who underwent HoLEP or mLC-HoLEP for BPH between January 2021 and May 2023. All clinical data were prospectively recorded in the preoperative and postoperative periods and retrospectively analyzed within the scope of the study. Ethical approval was obtained from the Eskişehir City Hospital Non-Interventional Clinical Research Ethics Committee (Decision Date: June 16, 2023; Decision No: ESH/GOEK 2023/41).

Patients aged ≥ 50 years with a prostate volume ≥ 35 mL, an International Prostate Symptom Score (IPSS) ≥ 12, LUTS refractory to medical therapy, and no history of prior prostate or urethral surgery were included in the study. Patients with a history of urethral stricture, neurogenic bladder, severe coagulopathy, active infection, clinically significant prostate cancer, or a follow-up period of less than 12 months were excluded.

After applying the exclusion criteria, 47 patients who underwent mLC-HoLEP were included in the study. The control group consisted of 48 patients who underwent standard HoLEP and were matched in a 1:1 ratio. Propensity score matching was performed using the nearest-neighbor method without replacement based on age, prostate volume, preoperative IPSS, and preoperative Qmax. Post-matching balance between the groups was assessed using the standardized mean difference (SMD), with an SMD < 0.10 considered indicative of adequate balance.

### Surgical technique

**Fig. 1:**
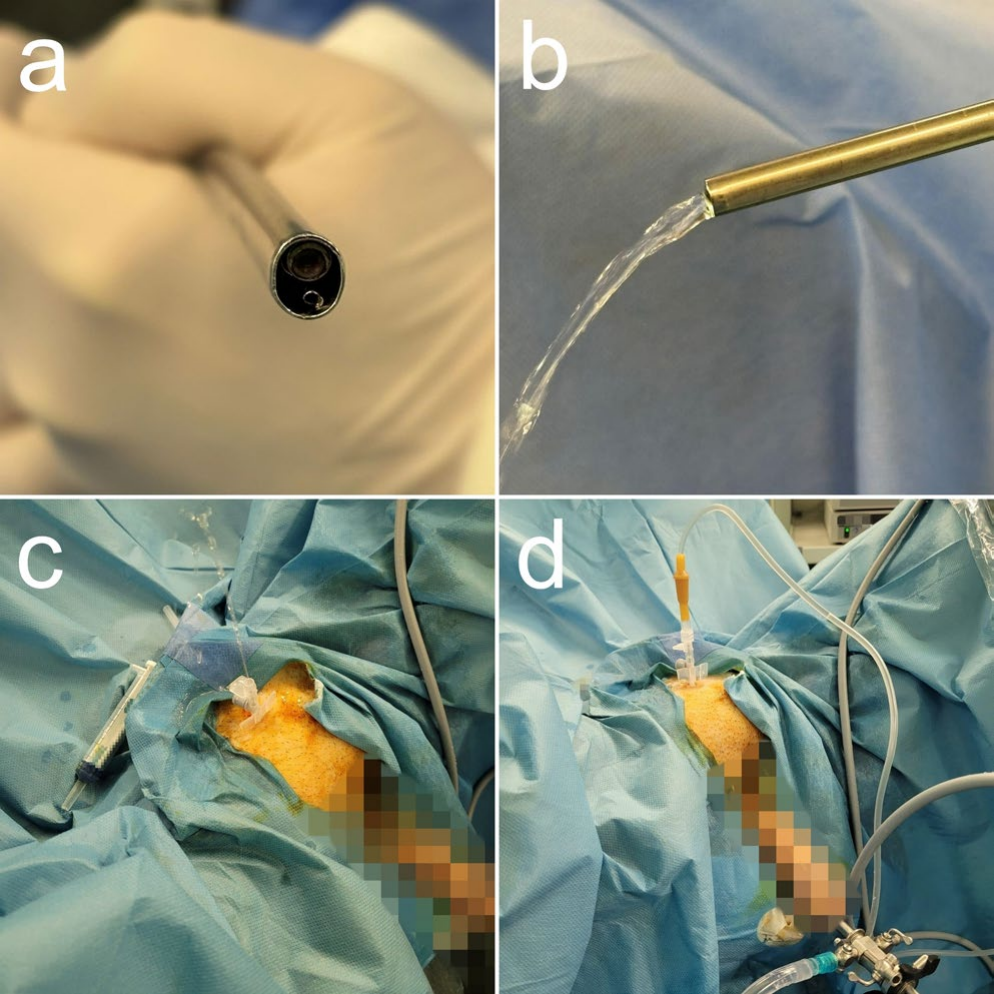
Intraoperative view of the equipment used in the modified low-caliber HoLEP (mLC-HoLEP) technique and suprapubic drainage. **a** Distal tip view of the 21 Fr internal urethrotomy sheath used for the mLC-HoLEP procedure. **b** Visualization of fluid flow within the sheath during one-way irrigation. **c** Intraoperative view of a 16 G cannula placed suprapubically for passive drainage. **d** Final configuration of the cannula integrated with the connection set to facilitate irrigation drainage

As dedicated MiLEP sets were not available at our center, a 21 Fr internal urethrotomy sheath was used for the mLC-HoLEP procedure. To ensure adequate continuous-flow irrigation–outflow during one-way irrigation, passive suprapubic bladder drainage was established using a 16 G cannula; the cannula was anchored to the abdominal wall using a sterile sponge and adhesive tape to minimize the risk of dislodgement (Fig. 1). After completion of the enucleation phase using the reduced-caliber sheath, the morcellation step was performed using a conventional 26 Fr morcelloscope. All procedures were performed by two surgeons who had each completed more than 100 HoLEP procedures before the study period. Enucleation was carried out using a Lumenis^®^ 120 W Ho:YAG laser (Lumenis Ltd., Yokneam, Israel) with settings of 1.5 J and 50 Hz (total power 75 W), employing the en bloc technique with early apical release. Morcellation was completed using a VersaCut^®^ morcellator (Lumenis Inc., Santa Clara, CA, USA).

### Data collection and follow-up

Patient data including age, comorbidities, prostate volume, duration of alpha-blocker use, preoperative and postoperative International Prostate Symptom Score (IPSS), uroflowmetry parameters, hemoglobin and creatinine levels, International Index of Erectile Function (IIEF) scores, operative time, irrigation volume, and length of hospital stay were recorded.

Postoperative follow-up evaluations at 1, 3, 6, and 12 months included assessment of continence status, urethral stricture formation, irritative symptoms, and the need for reintervention.

### Statistical analysis

Continuous variables were reported as mean ± standard deviation or median (minimum–maximum), while categorical variables were expressed as percentages (%). The normality of continuous variables was assessed using the Shapiro–Wilk test. Comparisons between the two groups were performed using the independent samples t-test or the Mann–Whitney U test, as appropriate. Categorical variables were compared using the chi-square test or Fisher’s exact test. All statistical analyses were conducted using the Statistical Package for Social Sciences (SPSS version 20.0 for Windows; Chicago, IL, USA).

For sample size calculation, the primary endpoint was defined as the rate of transient stress urinary incontinence observed at postoperative month 1. Based on the data reported by Taha et al. [10], the incontinence rate was assumed to be 42% in the standard HoLEP group and 15% in the mLC-HoLEP group. Using a two-sided test for comparison of two independent proportions with an α level of 0.05 and a power (1 − β) of 0.80, the minimum required sample size was calculated as 43 patients per group with a 1:1 allocation ratio.

Secondary endpoints were predefined as changes in IPSS, improvement in Qmax, total irrigation volume, development of urethral stricture, and other perioperative complications. The incontinence rates used for sample size estimation were based on relatively high early postoperative rates reported in the literature; however, it should be acknowledged that lower rates may be observed in contemporary clinical practice.

## Results

After application of the exclusion criteria, 48 patients who underwent standard HoLEP and 47 patients who underwent mLC-HoLEP were included in the study. There were no statistically significant differences between the groups in terms of age, body mass index (BMI), prostate volume, preoperative IPSS, Qmax, or postvoid residual urine (PVR) (Table 1).

**Table 1:** Comparison of demographic and preoperative data of patients undergoing HoLEP and m-HoLE

	HoLEP (*n* = 48)	mLC-HoLEP (*n* = 47)	*p*
Age (median, min–max)	68 (56–75)	67 (55–75)	0.345*
Height (cm) (median, min–max)	168 (152–180)	165 (150–180)	0.105*
Body weight (kg) (mean ± SD)	77.51 ± 14.79	79.78 ± 12.82	0.425****
Body mass index (median, min–max)	27.16 (18.99–34.02)	30.42 (21.53–34.02)	0.118*
Medical history (n, %)	28 (58.3%)	21 (44.7%)	0.183**
Hypertension	13 (27.1%)	7 (14.9%)	0.145**
DM	12 (25%)	15 (31.9%)	0.455**
COPD	8 (16.7%)	4 (8.5%)	0.232**
CAD	6 (12.5%)	3 (6.4%)	0.254***
Alpha blocker usage duration (weeks) (median, min–max)	16 (9–25)	16 (8–26)	0.558*
Alpha-blocker use (n, %)			0.600**
Doxazosin	17 (35.4%)	11 (23.4%)	
Alfuzosin	8 (16.7%)	9 (19.1%)	
Tamsulosin	10 (20.8%)	10 (21.3%)	
Silodosin	13 (27.1%)	17 (36.2%)	
Number of patients with an indwelling catheter (n, %)	4 (8.3%)	2 (4.3%)	0.349***
Preoperative PSA (ng/mL) (median, min-max)	3.07 (0.38–7.92)	3.04 (0.45–7.99)	0.970*
Prostate volume (g) (median, min–max)	75.5 (36–113)	66 (32–107)	0.173*
Preoperative IPSS (mean ± SD)	21.92 ± 5.06	21.62 ± 3.55	0.739****
Preoperative IIEF (median, min–max)	12 (5–20)	13 (7–22)	0.208*
Preoperative uroflow volume (mL) (median, min-max)	269 (148–392)	246 (157–394)	0.324*
Preoperative uroflow Qmax (mL/s) (mean ± SD)	8.18 ± 2.11	7.73 ± 1.97	0.277****
Preoperative residual urine volume (mL) (mean ± SD)	103.6 ± 21.03	98.81 ± 22.18	0.282****

*HoLEP* holmium laser prostate enucleation, *mLC-HoLEP* modified low-caliber HoLEP, *DM* diabetes mellitus, *COPD* chronic obstructive pulmonary disease, *CAD* coronary artery disease, *IPSS* International Prostate Symptom Score, *IIEF* International Erectile Function Index, *SD* standard deviation, *min* minimum, *max* maximum

*Mann Whitney U test

**Chi square test

***Fisher’s exact test

****Student T test

Evaluation of perioperative outcomes revealed no significant differences between the groups regarding operative time, enucleation time, morcellation time, or specimen weight (all *p* > 0.05). In contrast, the total irrigation volume was significantly higher in the standard HoLEP group (22.28 ± 3.12 L) compared with the mLC-HoLEP group (16.92 ± 2.19 L; *p* < 0.001). No significant difference was observed between the groups in terms of preoperative and postoperative hemoglobin drop (*p* = 0.392) (Table 2).

**Table 2:** Comparison of perioperative and postoperative data and early postoperative complications in patients undergoing HoLEP and m-HoLEP

	HoLEP (*n* = 48)	mLC-HoLEP (*n* = 47)	*p*
Operation time (min) (mean ± SD)	78.75 ± 6.52	76.89 ± 6.92	0.182**
Enucleation time (median, min–max)	34 (19–64)	37 (22–69)	0.161*
Morcellation time (mean ± SD)	16.27 ± 5.14	15.91 ± 5.17	0.750**
Intra-operative irrigation amount (l) (mean ± SD)	22.28 ± 3.12	16.92 ± 2.19	< 0.001**
Complications related to suprapubic drainage (n, %)	N/A	0 (0.0%)	–
Catheterization duration (days) (median, min–max)	2 (1–4)	1 (1–2)	0.366*
Length of stay (days) (median, min–max)	1 (1–2)	1 (1–2)	0.883*
Hemoglobin drop (mean ± SD)	− 1.21 ± 0.41	− 1.27 ± 0.34	0.392**
Creatinine change (median, min–max)	0.05 (− 0.13 to 0.12)	0.69 (− 0.13 to 2.15)	0.952*
Specimen weight (median, min–max)	47.5 (21–86)	43 (15–75)	0.212*
Enucleation efficiency (g/min) (mean ± SD)	1.33 ± 0.28	1.10 ± 0.27	< 0.001**
Postoperative total PSA (ng/mL) (median, min–max)	0.61 (0.05–2.18)	0.69 (0.13–2.15)	0.982*
Postoperative uroflow volume (mL) (median, min–max)	419 (312–600)	390 (300–591)	0.179*
Postoperative uroflow Qmax (mL/s) (mean ± SD)	23.12 ± 2.84	22.29 ± 3.97	0.249**
Postoperative residual urine (mL) (median, min–max)	42.5 (0–101)	36 (0–86)	0.190*
Postoperative IPSS (median, min–max)	9 (3–21)	9 (3–19)	0.598*
Postoperative IIEF (median, min–max)	12 (4–23)	13 (6–24)	0.351*
30-day emergency department visit rates (n, %)	2 (4.2%)	5 (10.6%)	0.368****
Postoperative complications (n, %)			
Early retention	8 (16.7%)	7 (14.9%)	0.813***
Hematuria	2 (4.2%)	2 (4.3%)	1.000****
Urinary tract infection	3 (6.3%)	3 (6.4%)	1.000****
Urge incontinence	4 (8.3%)	3 (6.4%)	1.000****
Stress incontinence	12 (25%)	5 (10.6%)	0.068***
Pad use at 1 month	12 (25%)	5 (10.6%)	0.068***
Pad use at 3 month	1 (2.1%)	1 (2.1%)	1.000****
Pad use at 12 month	1 (2.1%)	0 (0.0%)	1.000***
Late stress incontinence	1 (2.1%)	0 (0.0%)	1.000***
Urethral stricture	0 (0.0%)	0 (0.0%)	–
Recatheterization	1 (2.1%)	0 (0.0%)	1.000***
Orchitis	1 (2.1%)	0 (0.0%)	1.000***
Mea dilation	4 (8.3%)	0 (0.0%)	0.117****
Complication grade (n, %) according to the Clavien–Dindo classification			0.698***
Grade 1	12 (25%)	9 (19.1%)	
Grade 2	5 (10.4%)	5 (10.6%)	
Grade 3	0 (0.0%)	1 (2.1%)	

*HoLEP* holmium laser prostate enucleation, *mLC-HoLEP* modified low-caliber HoLEP, *IPSS* International Prostate Symptom Score, *IIEF* International Index of Erectile Function, *SD* standard deviation, *min* minimum, *max* maximum

*Mann Whitney U test

**Student T test

***Chi-square test

****Fisher’s Exact test

Enucleation efficiency, calculated as the ratio of enucleated tissue weight (grams) to enucleation time (minutes), was 1.10 ± 0.27 g/min in the mLC-HoLEP group and 1.33 ± 0.28 g/min in the standard HoLEP group (*p* < 0.001), indicating a statistically higher efficiency in the standard HoLEP group. Despite this difference, no significant differences were observed in functional outcomes, hemostasis parameters, or catheterization duration between the groups (Table 2).

Comparison of postoperative functional outcomes revealed no statistically significant differences between the HoLEP and mLC-HoLEP groups in terms of IPSS, Qmax, or PVR values (all *p* > 0.05). The mean postoperative Qmax was 23.12 ± 2.84 mL/s in the HoLEP group and 22.29 ± 3.97 mL/s in the mLC-HoLEP group, while postoperative IPSS values (median, min–max) were 9 (3–21) and 9 (3–19), respectively. No major complications related to suprapubic drainage were observed. Specifically, no cases of local hematoma, infection, urinary leakage, or inadequate drainage were detected (Table 2).

With respect to early postoperative complications, the incidences of urinary retention, hematuria, urinary tract infection, urgency, and fever were similar between the two groups (*p* > 0.05). Although early stress urinary incontinence and pad use during the first postoperative month were numerically higher in the HoLEP group, this difference did not reach statistical significance (*p* = 0.068). Continence rates at the third and twelfth postoperative months were comparable between the groups. Thirty-day emergency department visits occurred in 5 patients in the mLC-HoLEP group and 2 patients in the standard HoLEP group (*p* = 0.268). The majority of visits in the mLC-HoLEP group were related to transient irritative lower urinary tract symptoms and mild hematuria not requiring intervention; no patient in either group required re-hospitalization, blood transfusion, or return to the operating room within 30 days, and none of the emergency visits were attributable to suprapubic cannula-related complications. Likewise, no significant differences were observed regarding urethral stricture formation or the need for recatheterization (Table 2).

In both groups, Clavien–Dindo complication grades were predominantly grade 0–1, with no statistically significant difference between the groups (*p* = 0.698). Overall, both techniques demonstrated comparable safety and efficacy profiles, with a statistically significant difference observed only in favor of mLC-HoLEP with respect to total irrigation volume.

## Discussion

MiLEP is a minimally invasive approach described by de Figueiredo and Teloken that aims to reduce urethral trauma through the use of dedicated miniaturized instruments with diameters of 18.5–22 Fr and specialized continuous-flow irrigation–outflow systems [8]. Techniques referred to as mini-HoLEP in the literature are typically performed using standard 21–22 Fr resectoscopes, with morcellation often completed using conventional 26 Fr instruments [10, 11]. These approaches suggest that smaller-caliber sheaths may reduce early postoperative incontinence and urethral trauma. In a meta-analysis by Adhoni et al., surgical efficiency was comparable to standard HoLEP in cases using small-caliber resectoscopes, while the need for urethral dilation was significantly reduced [3]. Furthermore, the prospective MiLEP series by Barros Alves et al. demonstrated high early- and mid-term continence rates, and the study by Taha et al. reported lower irrigation volumes and reduced requirements for meatal dilation in the group treated with smaller-caliber sheaths [9, 10].

Consistent with previous studies, postoperative IPSS and Qmax values in the mLC-HoLEP group were comparable to those observed in the standard HoLEP group in our study [3, 9, 10]. This finding supports the notion that enucleation performed with smaller-caliber instruments does not compromise functional efficacy. Although early stress urinary incontinence and pad use were numerically lower in the mLC-HoLEP group, the difference between the groups did not reach statistical significance. Contemporary literature indicates that when miniaturized techniques are performed by experienced, high-volume surgeons, first-month incontinence rates for both techniques generally range between 4% and 6% [12]. Similarly low rates were observed in the present study, and differences of this magnitude are difficult to demonstrate statistically with the current sample size. Therefore, the absence of a statistically significant difference in early incontinence should not be interpreted as evidence of equivalence between techniques, but rather as a reflection of limited statistical power for this endpoint and the diminishing discriminatory value of early incontinence in current clinical practice.

In our study, the total irrigation volume was significantly lower in the mLC-HoLEP group compared with standard HoLEP. This difference should be considered not only a technical hydrodynamic outcome but also a potential clinical advantage. A lower irrigation volume may provide a safer perioperative fluid load, a reduced risk of hemodilution, and potentially more stable hemodynamics, particularly in elderly patients and those with cardiac comorbidities, heart failure, or sensitivity to fluid overload. In addition, reduced irrigation volume may theoretically lower the risks of hyponatremia, fluid absorption, and postoperative edema. However, the direct impact of this potential advantage on advanced clinical endpoints, such as cardiac complications or the need for intensive care, was not evaluated in the present study and should be confirmed by future prospective investigations. It is important to note that reduced irrigation volume alone cannot be interpreted as evidence of lower intravesical pressure (IVP), as IVP is determined by the dynamic balance between inflow rate and outflow resistance rather than total volume. While the suprapubic bladder cannula provides a passive outflow pathway that may facilitate pressure equilibration, IVP was not directly measured in this study; therefore, the proposed low-pressure advantage should be considered a physiologically plausible but unverified effect. Furthermore, fluid absorption was not formally evaluated, as perioperative body weight changes and irrigation fluid deficit were not systematically recorded; this represents a methodological limitation, and subclinical fluid absorption cannot be excluded. Future prospective studies should incorporate standardized perioperative weight measurements and targeted biochemical assessments as minimum requirements for reliable fluid absorption monitoring. Nevertheless, the current findings suggest that the modified technique may offer not only comparable efficacy but also a potential physiological benefit.

Regarding urethral stricture formation, Thai et al. compared 26 Fr and 28 Fr resectoscope sheaths and reported a lower stricture rate in the 26 Fr group [13]. Similarly, in the series by Ibis and Tokatli, no significant difference in stricture development was observed between the 22 Fr and 26 Fr groups [11]. In our cohort, urethral strictures were infrequent in both groups, with no statistically significant difference between them. These findings suggest that performing morcellation for a limited duration using a 26 Fr morcelloscope does not meaningfully increase the long-term risk of urethral stricture. Nevertheless, 12 months of follow-up is insufficient to draw definitive conclusions regarding urethral stricture, and longer-term evaluation is required before firm conclusions can be drawn.

Suprapubic bladder drainage allowed effective evacuation of irrigation fluid and may help to maintain lower intravesical pressure; however, intravesical pressure was not directly measured in our series; formal confirmation of this proposed physiological advantage therefore requires future prospective studies incorporating direct manometric assessment. Improved irrigation efficiency is further supported by previous studies; Wilhelm et al. demonstrated that continuous-flow irrigation–outflow systems enhance irrigation dynamics and contribute to reduced perioperative complications [14]. In our series, no major complications related to suprapubic drainage were observed. This is consistent with the large-cohort safety data reported by Hobbs et al. [15], who demonstrated a major complication rate of only 0.6% and no bowel injuries among 1000 patients undergoing suprapubic catheterisation; given that our technique uses a substantially less invasive 16G cannula rather than a formal cystostomy, the associated risk is expected to be lower still. This strategy was intended to facilitate passive outflow during one-way irrigation and may have contributed to adequate intraoperative visibility; however, this was not objectively measured. No cannula obstruction or dislodgement occurred. In obese patients in whom suprapubic cannula access to the bladder may be inadequate, drainage can be safely maintained using percutaneous cystostomy. Dean et al. [16] conducted a prospective randomized trial comparing 24 Fr and 28 Fr resectoscope sheaths in HoLEP and demonstrated that larger sheath size was associated with improved surgeon-rated irrigation flow and visibility (*p* < 0.001), higher rates of same-day trial of void, and shorter length of stay. Importantly, however, the authors reported no significant differences between groups in 90-day emergency visits, readmissions, complications, or functional outcomes, a finding consistent with our results. Furthermore, the Dean et al. [16] comparison was performed in a conventional closed HoLEP system without an independent drainage pathway, whereas our mLC-HoLEP technique incorporates a 16G suprapubic cannula providing continuous passive outflow. The overall fluid dynamics of our system therefore differ fundamentally from a simple comparison of sheath sizes, and the results of Dean et al. [16] cannot be directly extrapolated to our approach. The numerically higher 30-day emergency department visit rate observed in the mLC-HoLEP group (5 vs. 2 patients, *p* = 0.268) is noteworthy, although this difference did not reach statistical significance and should be interpreted with caution given the limited number of events. The majority of these visits were related to mild, self-limiting irritative lower urinary tract symptoms that did not require intervention. The underlying mechanism remains unclear, and no mechanistic inference can be drawn from the present data. Further prospective studies are required to better elucidate these findings.

The most distinctive aspect of our study is the successful replication of miniaturization without the use of dedicated MiLEP equipment, achieved by combining a routinely available 21 Fr internal urethrotomy sheath with suprapubic drainage. This approach eliminates the need for dedicated ultra-slim resectoscopes, specialized fiberoptic systems, and proprietary drainage sets, thereby removing equipment accessibility as a major barrier to minimally invasive enucleation. For centers that lack access to MiLEP technology but have established HoLEP infrastructure, this method offers a readily applicable and safe alternative. In this regard, the present study proposes not merely a technical modification but a practical clinical solution that may facilitate the wider dissemination of minimally invasive prostate surgery across diverse clinical settings.

Future prospective, multicenter studies with direct comparisons to a true MiLEP cohort are needed to more clearly define the impact of this modified technique on early continence outcomes, irrigation volume, intravesical pressure, cardiovascular safety, and long-term urethral stricture rates. In addition, the inclusion of cost-effectiveness analyses and patient-reported quality-of-life outcomes and evaluation of preoperative 5-alpha reductase inhibitor therapy on surgical planes will be critical for determining the potential role of this approach in clinical guidelines.

The main limitations of the present study include its retrospective design, single-center setting, lack of randomization, relatively small sample size, and the absence of a direct comparison with a true MiLEP group. Moreover, the follow-up period was limited to 12 months, which is insufficient to draw definitive conclusions regarding urethral stricture formation and reintervention rates, necessitating longer-term observation. Although both surgeons had completed more than 100 HoLEP procedures before the study period, recent data suggest that the HoLEP learning curve may continue beyond this threshold; therefore, surgeon experience and learning-curve effects should be considered when interpreting the findings. Intravesical pressure was not directly measured; therefore, the proposed low-pressure advantage remains physiologically plausible but unverified. Fluid absorption was not formally assessed, as perioperative body weight changes and irrigation fluid deficit were not systematically recorded, and subclinical fluid absorption cannot be excluded. Preoperative use of 5-alpha reductase inhibitors (5-ARI) was not systematically recorded as a separate variable, which may have influenced tissue characteristics and should be accounted for in future prospective studies. Furthermore, the hybrid nature of the technique—utilizing a reduced-caliber sheath for enucleation and a standard 26 Fr morcelloscope for the shorter morcellation phase—means that any reduction in urethral instrumentation time is partial rather than complete; parameters such as meatal dilation, urethral calibration, and exact duration of large-caliber instrumentation were not systematically recorded. Although enucleation efficiency was statistically lower in the mLC-HoLEP group (1.10 ± 0.27 vs. 1.33 ± 0.28 g/min; *p* < 0.001), this did not translate into clinically meaningful differences in postoperative outcomes, consistent with prior miniaturized enucleation literature.

## Conclusion

In this study, the mLC-HoLEP technique performed using a 21 Fr internal urethrotomy sheath combined with suprapubic drainage demonstrated functional efficacy (IPSS and Qmax) and a safety profile comparable to those of standard HoLEP. The significantly lower total irrigation volume observed with the modified technique represents an important finding that may confer a potential hemodynamic advantage in patients sensitive to fluid overload; however, direct intravesical pressure measurement was not performed, and confirmation of this physiological advantage awaits prospective investigation. In addition, suprapubic drainage was safely applied without procedure-related complications. The ability to perform this technique using commonly available endourological instruments without the need for dedicated MiLEP equipment renders it a resource-adapted technical modification representing an accessible surgical alternative, particularly for centers operating in resource-limited settings.

## Data Availability

The datasets used and/or analyzed during the current study are available from the corresponding author on reasonable request.
